# Vitamin E Is Superior to Vitamin C in Delaying Seedling Senescence and Improving Resistance in Arabidopsis Deficient in Macro-Elements

**DOI:** 10.3390/ijms21197429

**Published:** 2020-10-08

**Authors:** Zhong-Wei Zhang, Xin-Yue Yang, Xiao-Jian Zheng, Yu-Fan Fu, Ting Lan, Xiao-Yan Tang, Chang-Quan Wang, Guang-Deng Chen, Jian Zeng, Shu Yuan

**Affiliations:** College of Resources, Sichuan Agricultural University, Chengdu 611130, China; zzwzhang@126.com (Z.-W.Z.); Yang16970319@163.com (X.-Y.Y.); fgazelle@126.com (X.-J.Z.); stefanlife@126.com (Y.-F.F.); tlan@sicau.edu.cn (T.L.); wufanwf33@126.com (X.-Y.T.); zzwzhang@sicau.edu.cn (C.-Q.W.); gdchen@sicau.edu.cn (G.-D.C.); doplin@gmail.com (J.Z.)

**Keywords:** macro-element deficiency, senescence, vitamin C, vitamin E, oxidative stress

## Abstract

Nitrogen (N), phosphorus (P), and potassium (K) are three essential macro-elements for plant growth and development. Used to improve yield in agricultural production, the excessive use of chemical fertilizers often leads to increased production costs and ecological environmental pollution. Vitamins C and E are antioxidants that play an important role in alleviating abiotic stress. However, there are few studies on alleviating oxidative stress caused by macro-element deficiency. Here, we used Arabidopsis vitamin E synthesis-deficient mutant *vte4* and vitamin C synthesis-deficient mutant *vtc1* on which exogenous vitamin E and vitamin C, respectively, were applied at the bolting stage. In the deficiency of macro-elements, the Arabidopsis chlorophyll content decreased, malondialdehyde (MDA) content and relative electric conductivity increased, and reactive oxygen species (ROS) accumulated. The mutants *vtc1* and *vte4* are more severely stressed than the wild-type plants. Adding exogenous vitamin E was found to better alleviate stress than adding vitamin C. Vitamin C barely affected and vitamin E significantly inhibited the synthesis of ethylene (ETH) and jasmonic acid (JA) genes, thereby reducing the accumulation of ETH and JA that alleviated the senescence caused by macro-element deficiency at the later stage of bolting in Arabidopsis. A deficiency of macro-elements also reduced the yield and germination rate of the seeds, which were more apparent in *vtc1* and *vte4,* and adding exogenous vitamin C and vitamin E, respectively, could restore them. This study reported, for the first time, that vitamin E is better than vitamin C in delaying seedling senescence caused by macro-element deficiency in Arabidopsis.

## 1. Introduction

During plant growth, metabolic activities, protein synthesis, vegetative growth, reproductive growth, and increased yield depend on an adequate supply of nitrogen (N) [1]. Insufficient N not only limits the growth of plants but also affects their morphogenesis [2]. N deficiency can also cause a decrease in photosynthetic efficiency, which in turn leads to a decrease in chlorophyll content and ribulose-1,5-bisphosphate oxygenase (Rubisco) activity [3]. Phosphorus (P) participates in the growth, development, and reproduction of plants, and plays an important role in life activities such as energy metabolism, photosynthesis, and signal transmission [4]. In the long-term evolutionary process, plants have developed more sophisticated strategies to better adapt to P starvation, including changes in the structure of the root system (reducing the growth of primary roots and promoting more lateral roots and root hairs) [5]. The formation of phosphate transporters increases the expression of the P transporter gene that leads to an induction of the secretion of organic acids, RNases, acid phosphatases (APases), and the accumulation of starch and anthocyanins, thereby enhancing plants’ resistance to P deficiency [6]. Potassium (K), a major element necessary for plants to complete their life cycles, also participates in many physiological processes. As an osmotic regulating substance, K participates in the rapid expansion of cells and promotes growth [7,8] and plays an important role in the transport of solutes through the phloem [9]. The transport process of sucrose solute from shoot to root and sink tissue in fruits requires K [10]. Wang and Wu [11] found that K is involved in the transmission of stress signals. For example, K deficiency induces the production of reactive oxygen species (ROS), affects resistance to disease and insect pests, and affects auxin synthesis [12]. The optimization of potassium nutritional status is very important for reducing the stress of biotic and abiotic stress [7]. Oxidation caused by ROS, such as superoxide ion (O_2_^−^), hydrogen peroxide (H_2_O_2_), hydroxyl radicals (•OH), and singlet oxygen (^1^O_2_) in biotic and abiotic stresses, is the most common, and those oxidative stresses can cause significant damage to cells [13]. When plants are deficient in macro-elements (N, P, K), they will activate the antioxidant system as an abiotic stress response and cause a series of redox processes [14].

Hormones are very important for the regulation of senescence. On the one hand, environmental factors affect leaf senescence mainly due to the plant’s responses by hormones ethylene (ETH) and jasmonic acid (JA) [15,16]. On the other hand, the synergistic effects of various hormones can promote or inhibit leaf senescence [17,18,19,20,21,22,23]. The hormone JA is key in mediating plants’ responses to environmental factors and endogenous signal substances, and participates in a series of physiological activities such as plant growth, development, maturation, and senescence [15]. The JA pathway plays an important regulatory role in the process of plant senescence and yellowing, and regulates the accumulation of starch in plant photosynthesis and the expression of senescence-related genes [17]. During senescence, the expression of JA synthesis-related genes is up-regulated [18]. The hormone ETH is gaseous in nature. For normal-growing leaves, treatment with a very low concentration of ETH can induce shedding, and treatment with ETH inhibitor can delay leaf senescence [16]. A variety of biotic and abiotic stresses stimulate the massive synthesis of ethylene through transcriptional activation of the *ASC* (*1-aminocyclopropane-1-carboxylic acid synthase*) gene [19,20]. Gene *EIN2* (*ethylene insensitive 2*) is a core member of the ethylene-signaling pathway that plays a role in the coordinated control of leaf senescence by JA [21]. The ETH response factor *EIN3* acts on the downstream of EIN2. EIN3 can bind to the upstream of the *ERF1* (*ethylene response factor 1*) promoter to regulate its transcription [22,23].

The content of vitamin E in plants is generally considered to be related to maturity and senescence. Studies have found that the old leaves of Arabidopsis plants contain high levels of *α-* and *γ*-tocopherols [24]. In lilies, the content of vitamin E increases as the petals age [25]. During senescence, plants face severe oxidative stress, leading to the increase in fatty acid free radicals. Increasing the content of vitamin E during senescence may be a strategy for plants to reduce stress [24]. At the same time, as a fat-soluble non-enzymatic antioxidant, vitamin E can effectively eliminate the production of ROS (mainly including ^1^O_2_ and •OH) by cooperating with other antioxidants [26]. Under stress conditions, the biosynthesis of vitamin E increases and provides better antioxidant protection by limiting the production of ROS [27].

Vitamin C is the most abundant and widely available water-soluble cellular antioxidant in plants [28]. Vitamin C can directly scavenge ROS or act as a substrate of ascorbate peroxidase (APX) to scavenge H_2_O_2_, allowing plants to avoid oxidative damage [29]. Moreover, vitamin C plays an important role in plant growth, development, and stress responses [30,31]. It is a cofactor for many enzymes, controls cell division, and affects cell expansion. It is also a regulator of plant senescence [32].

Vitamin E will irreversibly produce quinones and epoxides in the process of removing ROS, and chromogen alkoxy (TO•) will be produced during the conversion of lipid peroxy radicals (LOO•) [33]. With the participation of vitamin C through the ascorbic acid-glutathione cycle, these can be returned to vitamin E [34]. In addition, when plants are subjected to oxidative stress, vitamin E can effectively eliminate the production of ROS (mainly including ^1^O_2_ and •OH) by cooperating with other antioxidants (such as ascorbic acid) [35,36]. It can be seen that both vitamin E and vitamin C, as antioxidants, can remove ROS in plants and help plants resist oxidative damage under adversity stress. They are similar in function. Subtractive hybridization experiments have shown that a lack of N, P, and K can induce an upregulation of the *GGR* (geranylgeranyl reductase) gene in rape, which encodes a key enzyme involved in vitamin E synthesis [37]. This indicates that the deficiency of macro-elements may activate the antioxidant system in plants, such as increasing the expression of vitamin E in plants to resist a series of oxidative damage caused by macro-element deficiency [37].

At present, there are few studies on the mechanism of senescence caused by a deficiency in macro-elements. Vitamin C and vitamin E, as two kinds of antioxidants, are effective at alleviating oxidative stress and delaying senescence. However, no systematic comparison has been made between them. Therefore, in this study, *vtc1* and *vte4* were grown under a deficiency of macro-elements, and exogenous vitamin C and vitamin E were added as treatments. The results showed that both vitamin E and vitamin C could defer senescence caused by the deficiency of macro-elements. Vitamin E is superior to vitamin C in delaying senescence and improving plant resistance.

## 2. Results

### 2.1. Vitamin E and Vitamin C Defer Senescence Caused by the Deficiency of Macro-Elements in Arabidopsis

In order to explore the effects of vitamin C and vitamin E on the growth of mature Arabidopsis under macro-element deficiency, we first cultivated the experimental materials until bolting (25 days). Columbia ecotype Arabidopsis was used as the wild-type (WT), and *vtc1* and *vte4* were vitamin C and vitamin E synthetic-deficient mutants, respectively. In addition, 5 mM of vitamin E (WT+VE) and vitamin C solution (WT+VC) was sprayed on the WT every three days after bolting, representing the treatment of adding exogenous vitamin E and vitamin C. A part of each was removed to continue normal growth for the control (CK), and the rest were divided into three parts for the treatment of macro-element deficiency: deficiency of nitrogen (DN), deficiency of phosphorus (DP), and deficiency of potassium (DK).

**Figure 1:**
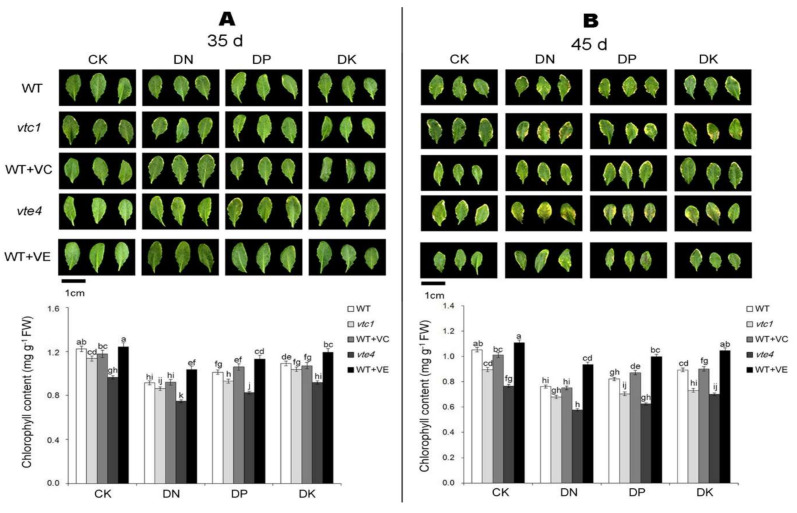
Leaf phenotypes and chlorophyll contents of *Arabidopsis thaliana* at the 35th day (**A**) and the 45th day (**B**) after germination. CK, DN, DP, DK represent normal, deficiency of nitrogen, deficiency of phosphorus, and deficiency of potassium treatments, respectively. *vtc1* and *vte4* are vitamin C and vitamin E synthetic deletion mutants, respectively. WT+VC and WT+VE represent treatments of 5 mM exogenous vitamin C and vitamin E, respectively, to the wild-type (WT) plants. Bar = 1 cm. FW, fresh weight. The data represent average values ± SEM (*n* = 3). Different small letters show significant differences (*p* < 0.05).

**Figure 2:**
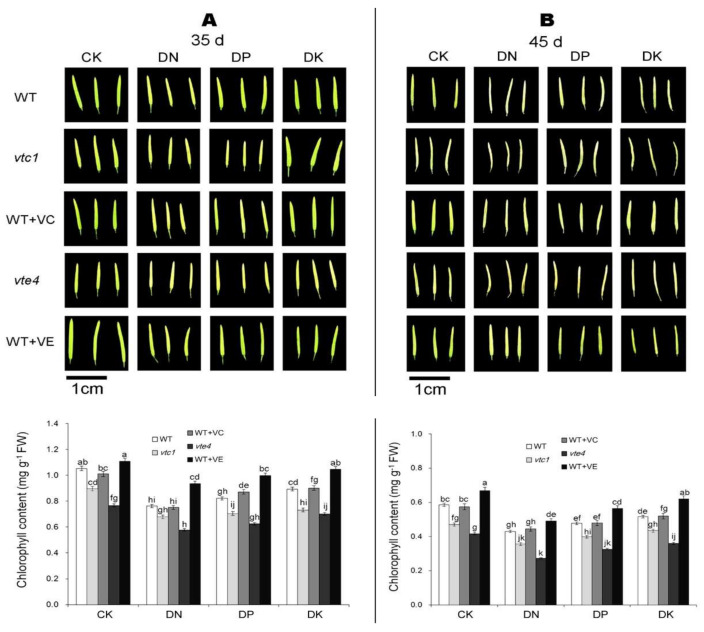
Seed pod phenotypes and chlorophyll contents of *Arabidopsis thaliana* at the 35th day (**A**) and the 45th day (**B**) after germination. CK, DN, DP, DK represent normal, deficiency of nitrogen, deficiency of phosphorus, and deficiency of potassium treatments, respectively. *vtc1* and *vte4* are vitamin C and vitamin E synthetic deletion mutants, respectively. WT+VC and WT+VE represent treatments of 5 mM exogenous vitamin C and vitamin E, respectively, to the wild-type (WT) plants. Bar = 1 cm. FW, fresh weight. The data represent average values ± SEM (*n* = 3). Different small letters show significant differences (*p* < 0.05).

After 10 days of macro-element deficiency (the 35th day), the leaves and seeds of Arabidopsis turned yellow and the chlorophyll content decreased (Figure 1A and Figure 2A). Under the CK condition, the degree of yellowing was the lowest; under DN treatment, the yellowing was the most prominent, followed by DP and DK treatment. Mutant *vte4* is the most affected by the deficiency of macro-elements where the chlorophyll content is the lowest. The yellowing is alleviated in the wild-type materials treated with an exogenous addition of vitamin E (WT+VE). The level of vitamin C apparently had less effect on leaf yellowing and senescence caused by the deficiency of macro-elements because the senescence process of *vtc1* and materials treated with exogenous vitamin C is the same as that of the WT (Figure 1A and Figure 2A).

After 20 days of macro-element deficiency (the 45th day), the senescence was further aggravated (Figure 1B and Figure 2B). Similar to the 10 days of deficiency of macro-elements, the degree of leaf yellowing in each treatment was DN>DP>DK>CK. Different from the result obtained after 10 days, after a longer period of stress, the leaves and seeds of *vtc1* turned yellow and the chlorophyll was degraded. Although the vitamin E and vitamin C synthetic-deficient mutants *vte4* and *vtc1*, respectively, had more severe stress than WT; an exogenous addition of vitamin E can only alleviate the stress caused by the deficiency of macro-elements (Figure 1A and Figure 2A).

### 2.2. Vitamin E and Vitamin C Relieve Oxidative Stress Caused by Macro-Element Deficiency by Removing Reactive Oxygen Species (ROS)

**Figure 3:**
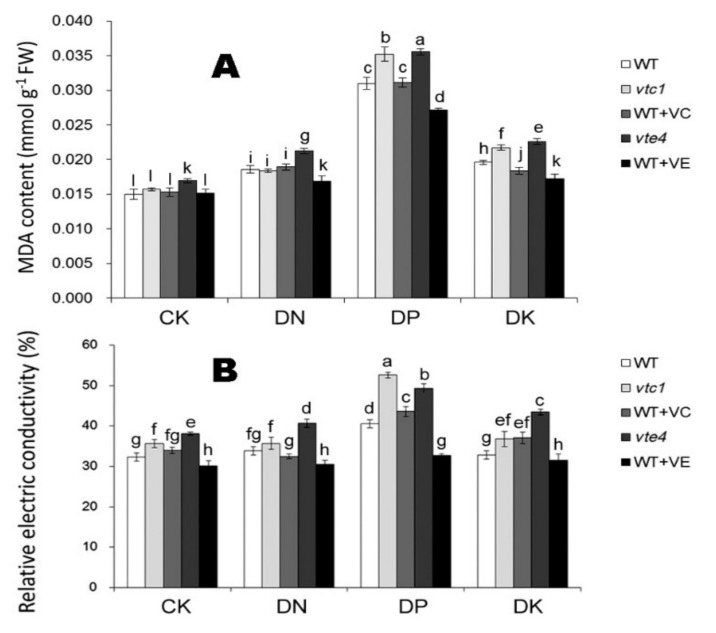
Malondialdehyde (MDA) content (**A**) and relative electric conductivity (**B**) of 45-day-old seedlings (20 days of nutrient deficiency treatments). CK, DN, DP, DK represent normal, deficiency of nitrogen, deficiency of phosphorus, and deficiency of potassium treatments, respectively. *vtc1* and *vte4* are vitamin C and vitamin E synthetic deletion mutants, respectively. WT+VC and WT+VE represent treatments of 5 mM exogenous vitamin C and vitamin E, respectively, to the wild-type (WT) plants. FW, fresh weight. The data represent average values ± SEM (*n* = 3). Different small letters show significant differences (*p* < 0.05).

After 20 days of macro-element deficiency, the content of malondialdehyde (MDA) in Arabidopsis under DN, DP, DK treatments was significantly higher than that under CK conditions. Under DP treatment, the MDA content was the highest, followed by DP and DK treatments. The *vte4* was most affected by the deficiency of macro-elements where the MDA content was the highest. Adding exogenous vitamin E effectively alleviated the oxidative damage caused by the stress, which is evident by the decrease in MDA content and electric conductivity (Figure 3).

In the *vtc1* mutant, the MDA content was similar to that of the WT when the degree of oxidative damage is low (DN condition), indicating that the reduction in vitamin C synthesis did not aggravate the stress of Arabidopsis under conditions of macro-elements deficiency, and when the stress was more severe (DP and DK condition), the MDA content was higher than the WT. Different from vitamin E, adding exogenous vitamin C did not alleviate the damage caused by deficiency (Figure 3).

**Figure 4:**
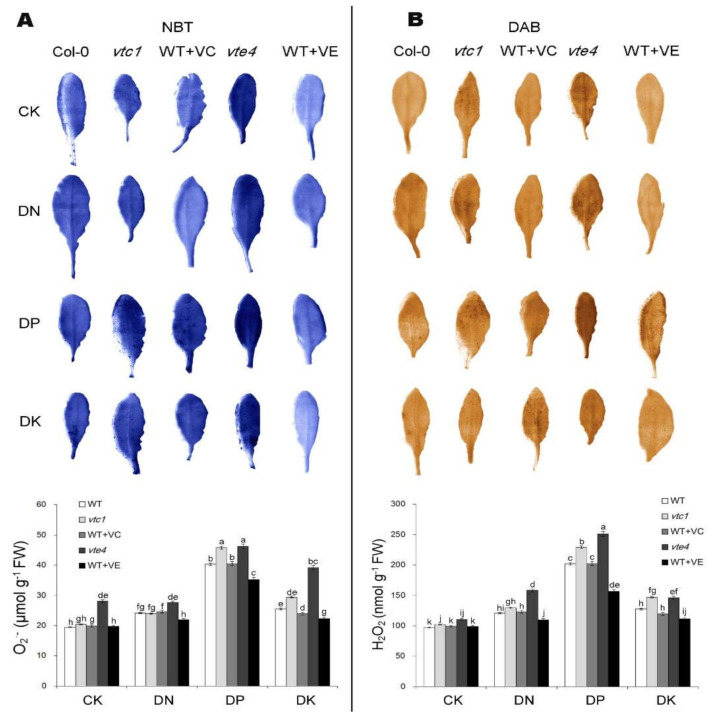
O_2_^−^ (**A**) and H_2_O_2_ (**B**) accumulation levels in 45-day-old seedlings (20 days of nutrient deficiency treatments). Histochemical assays for superoxide anion radicals (O_2_^−^) and H_2_O_2_ were performed by nitro-blue tetrazolium (NBT) and 3,3’-diamino-benzidine (DAB) staining, respectively. Quantitative data are presented below the staining images. CK, DN, DP, DK represent normal, deficiency of nitrogen, deficiency of phosphorus, and deficiency of potassium treatments, respectively. *vtc1* and *vte4* are vitamin C and vitamin E synthetic deletion mutants, respectively. WT+VC and WT+VE represent treatments of 5 mM exogenous vitamin C and vitamin E, respectively, to the wild-type (WT) plants. FW, fresh weight. The data represent average values ± SEM (*n* = 3). Different small letters show significant differences (*p* < 0.05).

To more intuitively reflect the oxidative damage caused by the deficiency of macro-elements, we selected Arabidopsis leaves after 20 days of macro-element deficiency treatment. The selected leaves were measured for the production levels of two major ROS species, H_2_O_2_ and O_2_^Ȓ^, under macro-elements deficiency stress, by histochemical staining using nitro-blue tetrazolium (NBT) and 3,3’-diamino-benzidine (DAB), respectively (Figure 4). The *vte4* mutant had the highest accumulation of H_2_O_2_ and O_2_^−^ with the most severe oxidative damage, and after the addition of exogenous vitamin E, the ROS level became lower than the WT (Figure 4).

**Figure 5:**
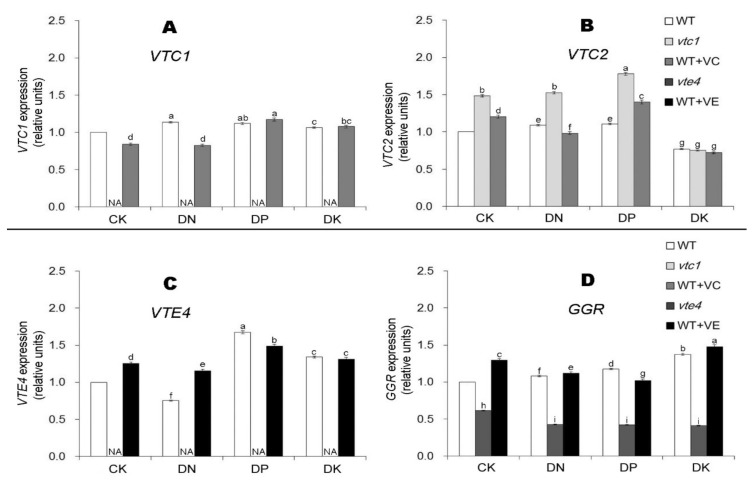
Relative expression of vitamin C synthetic genes *VTC1* (**A**) and *VTC2* (**B**) and vitamin E synthetic genes *VTE4* (**C**) and *GGR* (**D**) in 45-day-old seedlings (20 days of nutrient deficiency treatments). CK, DN, DP, DK represent normal, deficiency of nitrogen, deficiency of phosphorus, and deficiency of potassium treatments, respectively. *vtc1* and *vte4* are vitamin C and vitamin E synthetic deletion mutants, respectively. WT+VC and WT+VE represent treatments of 5 mM exogenous vitamin C and vitamin E, respectively, to the wild-type (WT) plants. VTC1, Vitamin C defective 1; VTC2, Vitamin C defective 2; VTE4, Vitamin E defective 4; GGR, Geranylgeranyl Reductase. The specific gene expression levels are represented as the percentages relatively to *ACTIN7* expression levels. The expression level of WT in the control sample (CK) was normalized into “1.0”. The data represent average values ± SEM (*n* = 3). Different small letters show significant differences (*p* < 0.05).

Vitamin E and vitamin C, as antioxidants, can remove ROS in plants and relieve the damage caused by oxidative stress. In *vtc1* mutant, the expression of *VTC2* is induced. Adding exogenous vitamin C inhibited the expression of *VTC1* under CK and DN conditions and induced the expression of *VTC1* and *VTC2* under DP and DK conditions (Figure 5A,B). In *vte4*, the expression of *GGR* was significantly inhibited and the expression was significantly lower than that of the WT. Under CK, DN, and DK conditions, adding exogenous vitamin E induced the expression of *VTE4* and *GGR*. In contrast, under DP conditions, adding exogenous vitamin E inhibited the expression of *VTE4* and *GGR* (Figure 5C,D).

### 2.3. Vitamin E Inhibits the Expression of Ethylene and Jasmonic Acid under Macro-Element Deficiency

**Figure 6:**
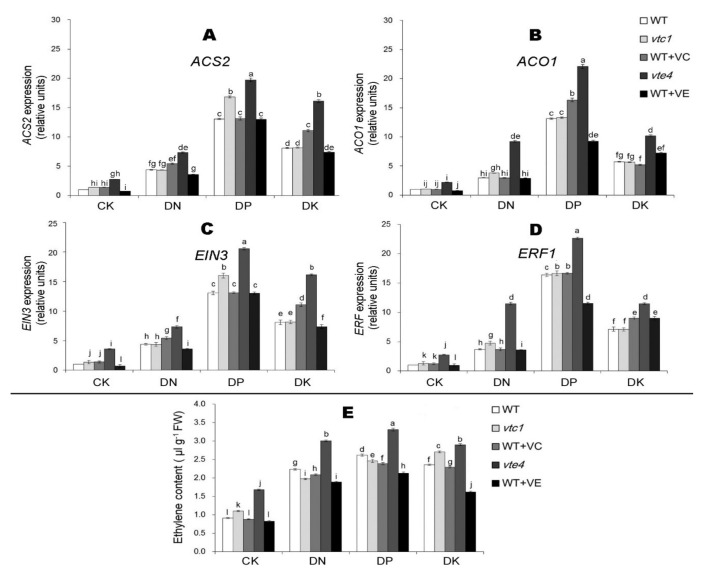
Relative expression of ethylene synthetic genes *ACS2* (**A**) and *ACO1* (**B**) and ethylene-signaling genes *EIN3* (**C**) and *ERF1* (**D**) and ethylene contents (**E**) in 45-day-old seedlings. ACS2, 1-Aminocyclopropane-1 -Carboxylic acid Synthase 2; ACO1, 1-Aminocyclopropane-1-Carboxylate Oxidase 1; EIN3, Ethylene Insensitive 3; ERF1, Ethylene Response Factor 1. The expression level of WT in the control sample (CK) was normalized into “1.0”. The data represent average values ± SEM (*n* = 3). Different small letters show significant differences (*p* < 0.05).

**Figure 7:**
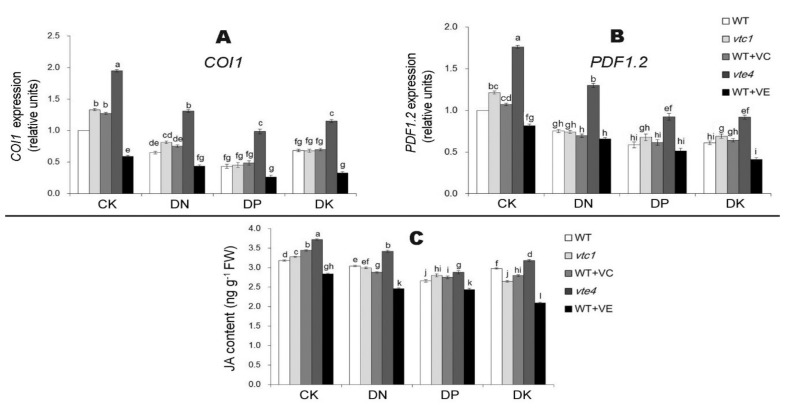
Relative expression of jasmonic acid synthetic gene *COI1* (**A**) and jasmonic acid-signaling gene *PDF1.2* (**B**) and jasmonic acid contents (**C**) in 45-day-old seedlings (20 days of nutrient deficiency treatments). COL1, CONSTANS-Like 1; PDF1.2, Plant Defensin 1.2. The expression level of WT in the control sample (CK) was normalized into “1.0”. The data represent average values ± SEM (*n* = 3). Different small letters show significant differences (*p* < 0.05).

We tested the ethylene content and its related gene expression levels in *Arabidopsis thaliana* after 20 days of macro-element deficiency. The results showed that the ethylene content, the expression level of ethylene synthesis-related genes *ACS2*, *ACO1*, and signaling pathway-related genes *EIN3* and *ERF1* under DN, DP, and DK treatments were higher than in CK conditions. The expression level was the highest under DP treatment, followed by DK and DN treatments. The *vte4* had the highest ethylene content and related gene expression levels, and the ethylene content after an exogenous addition of vitamin E treatments was lower than the WT. The level of vitamin C appeared to have little effect on the expression level of ethylene, because the senescence process of *vtc1* and materials treated with exogenous vitamin C was the same as that of the WT (Figure 6). After 20 days of macro-element deficiency treatment, the content of the other hormone JA and the expression level of its related genes *COI1* and *PDF1.2* were induced by stress (expression level DP>DK>DN>CK). The expression level of JA in *vtc1* and the materials treated with exogenous vitamin C were consistent with that of the WT, while in *vte4* it was significantly higher than that of the WT. An exogenous addition of vitamin E reduced the expression level of JA (Figure 7).

### 2.4. Vitamin E and Vitamin C Reverse the Yield Reduction of Arabidopsis under Macro-Element Deficiency

**Figure 8:**
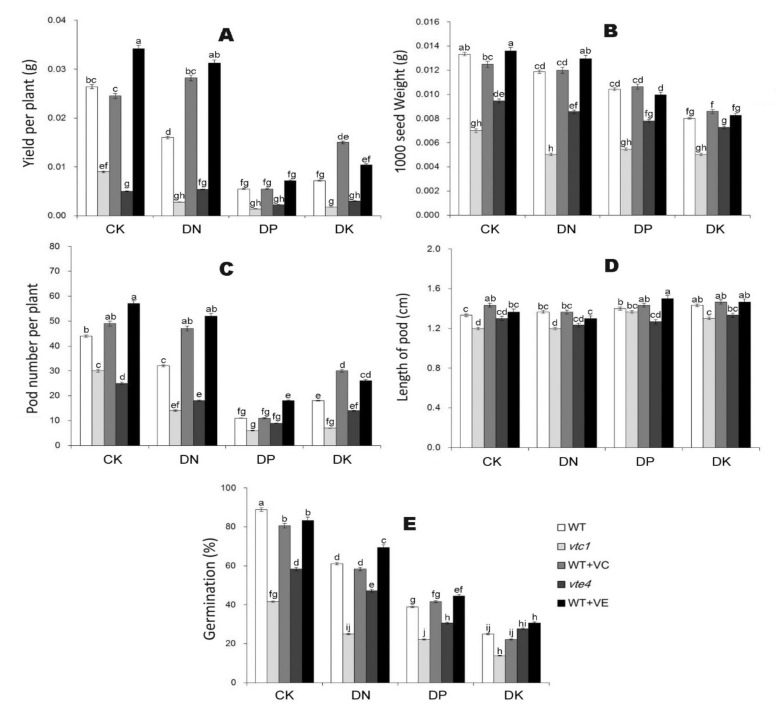
Yield per plant (**A**), 1000 seed weight (**B**), pod number per plant (**C**), length of pod (**D**), and seed germination rate (**E**) of Arabidopsis plants after macro-element deficiency treatments. CK, DN, DP, DK represent normal, deficiency of nitrogen, deficiency of phosphorus, and deficiency of potassium treatments, respectively. *vtc1* and *vte4* are vitamin C and vitamin E synthetic deletion mutants, respectively. WT+VC and WT+VE represent treatments of 5 mM exogenous vitamin C and vitamin E, respectively, to the wild-type (WT) plants. The data represent average values ± SEM (*n* = 3). Different small letters show significant differences (*p* < 0.05).

The Arabidopsis was cultivated for 21 days and treated with DN, DP, and DK treatment, and samples were taken after the seeds were mature. Arabidopsis under DP conditions had the lowest yield, followed by DK, and DN. The yield in *vtc1* and *vte4* was lower than that in the WT, while adding exogenous vitamin C and vitamin E significantly increased the yield, and vitamin E had a more potent effect on increasing production (Figure 8A). The results showed that the seed weight DK<DP<DN<CK (Figure 8B), the number of seed pods per plant DP<DK<DN<CK (Figure 8C), and the length of seed pods had no effect on the yield (Figure 8D). The Arabidopsis seeds under the different treatments were counted, and it was found that the germination rate of the seeds is related to their growth status before harvest. Seed germination rate was DK<DP<DN<CK, and the seed germination rate of *vtc1* and *vte4* was significantly lower than WT, and adding exogenous vitamin C and vitamin E did not change the germination rate of seeds significantly (Figure 8E).

## 3. Discussion

Studies have shown that the lack of N, P, and K elements in mulberry will activate the antioxidant system and cause a series of redox processes [38]. When plants are under DK stress, ROS in plant cells increase. The lack of vitamin E and vitamin C can lead to increased oxidative damage in *Arabidopsis thaliana* [39,40], and the damage is more serious under DK conditions [11,41,42]. The lack of vitamin E appears earlier than the lack of vitamin C (Figure 1). It may be due to the yellowing and senescence caused by vitamin C deficiency that it takes longer to manifest [43]. The alleviation of macro-element deficiency mainly relies on endogenous vitamin C—an external addition of vitamin C has no obvious effect [29,44]. Contrastingly, an exogenous addition of vitamin E showed an alleviating effect to the senescence. It may because that vitamin E is a strong antioxidant, which can reduce the degree of oxidative damages suffered by plasma and chloroplasts during the senescence [45,46].

In this study, vitamin E eliminated ROS and improved Arabidopsis resistance better than vitamin C. Studies with mammal cells have shown that the IC50 of vitamin E to eliminate H_2_O_2_ is 690 μM, while that of vitamin C is 860 μM, indicating that vitamin E has strong antioxidant properties than vitamin C [47,48], although the data in plant cells are still lacking.

As an important component of chlorophyll molecules, nitrogen has an important influence on chlorophyll synthesis and chloroplast development. It regulates photosynthesis by influencing chloroplast development, chlorophyll synthesis, and photo-cooperative enzyme activity [49]. In this study, under DN conditions, Arabidopsis chlorophyll synthesis was hindered; therefore, a loss of green was most apparent in the leaf and pod.

We found that the seed weight and germination rate were the lowest under DK conditions, although the oxidative stress suffered by plant was not the most severe. DK stress can indirectly affect photosynthesis by affecting the transport of photosynthetic products. Research by Zhong et al. [50] showed that DK stress reduced the yield of grafted watermelon, which was manifested by a decrease in the weight of each fruit, and the total soluble solids, sucrose, total soluble sugar, vitamin C, lycopene, and β-carotene in the fruit. DK mainly affects watermelon fruit quality through metabolic processes and pathways. Among them, the K channel gene *Cla020934* and the lycopene synthesis gene *Cla009122* are significantly down-regulated, indicating that DK affects watermelon fruit quality at the transcription level [50]. Tomato fruit quality is also affected by K because the early response to K deficiency is related to the decrease in photosynthesis rate and the transport of assimilate from the source leaves [51]. K participates in the transportation of photosynthetic products, and potassium deficiency causes the transportation of sucrose to be blocked and accumulate in the leaves. Studies have found that K deficiency affects the assimilation output of cotton leaves, leading to the accumulation of sugar at the source. The accumulation of sugar in the leaves over-compensates for the lack of K in the tissue in terms of permeability, while the sink organs are in a hungry state, and the photosynthetic rate of the leaves is also reduced [52]. ROS mainly come from photosynthetic electron transport [38], and thus severe oxidative damages under DK conditions could be explained. Because of the growth arrest induced by DK treatment, the quality of seeds decreased and the accumulated nutrients were insufficient, so the seed germination showed the most significant decrease.

The yield per plant and pod number per plant decreased under DN, DP, and DK treatments, and the DP had the lowest of the three (Figure 8A,C), indicating that DP treatment decreased the seeding rate the most significantly. DP leads to a phenotype of less branching or less tillering, and therefore may affect pod development [7,9]. Interestingly, the yield per plant and pod number per plant of the seedlings treated with exogenous vitamin E were higher than those of the seedlings without vitamin E treatment (Figure 8A,C). In other words, vitamin E may promote pod development or fertilization. However, the roles of P and vitamin E in Arabidopsis fertilization and pod development have not been well studied, and so require more investigations.

Nevertheless, all declines in yield and germination induced by macro-element deficiencies could be partly reversed by the exogenous vitamin E treatment. Vitamin E plays a key role in limiting non-enzymatic lipid oxidation during Arabidopsis seed storage, germination, and early seedling development [40].

In seedlings, ETH-signaling has been shown to reduce ROS accumulation to protect seedlings against salt stress and photo-oxidative damage by activating antioxidant enzyme system and enhancing antioxidant contents [16,53]. Besides this stress resistant signaling pathway, a study has shown that ETH regulates vitamin C synthesis by inhibiting the expression of *ABSCISIC ACID INSENSITIVE 4* (*ABI4*), promoting the transcription of *VTC2* in Arabidopsis and vitamin C biosynthesis, and inhibiting the accumulation of ROS in *Arabidopsis* seedlings [54]. However, in this study, the accumulation of ROS activated the expression of ETH signals in senescent leaves, indicating that there are differences between ethylene and ROS signals in seedlings and mature Arabidopsis plants.

An ethylene-signaling mutant *ein3* delayed the water stress-related increase in vitamin E and resulted in a 30% reduction in the levels of antioxidants, compared with the wild-type [55]. While an over-produced ethylene mutant, *eto1-1,* showed a 5-fold increase in vitamin E contents during leaf aging [55]. Thus, the perception and signal transduction of ETH may be involved in the regulation of vitamin E biosynthesis. On the contrary, here we showed that exogenous vitamin E repressed ethylene synthetic gene and ethylene-signaling gene expression, which might be attributed into its strong antioxidant capacity (see Discussion below).

There is little research on the relationship between JA and vitamin C and vitamin E. Both JA and ETH are related to senescence [15,16]. In this study, the expressions of ETH and JA are basically synchronized, indicating that ETH and JA may have similar signaling pathways in response to macro-element deficiency. When plants suffer from oxidative stress caused by a deficiency of macro-elements (or during senescence), ROS may function as a second messenger to induce JA and ETH biosynthesis [15,16]. Vitamin C and vitamin E are activated as antioxidants to remove ROS in plants, to avoid the accumulation of ETH and JA, and to delay the senescence of Arabidopsis. As an intermediate signaling molecule, ROS link vitamin E and vitamin C with ETH and JA, which reacts to senescence to form a chain-signaling pathway. In this process, vitamin E is faster and more effective than vitamin C in the removal of ROS.

## 4. Materials and Methods

### 4.1. Plant Materials and Growth Conditions

*Arabidopsis* mutants were soaked with 2 mg/L gibberellin solution, vernalized for 2 days under dark conditions at 4 °C, and then sown in pots containing vermiculite. Then the seedlings were grown in a culture room (temperature 23 °C; humidity 70%; light intensity 100 μmol·m^−2^·s^−1^; photoperiod 16 h light/8 h dark). A 1/2 MS nutrient solution (10 mM NH_4_NO_3_, 9.4 mM KNO_3_ and 0.5 mM KH_2_PO_4_) was irrigated every 3 days. After seedlings bolting (the 25th day), the treatments of macro-element deficiency were started by replacing the 1/2 MS nutrient solution with nutrient deficiency solutions. For the N deficiency (DN) treatment, NH_4_NO_3_ was removed and 9.4 mM KNO_3_ was replaced by 9.4 mM KCl; for the of P-deficient (DP) treatment, 0.5 mM KH_2_PO_4_ was replaced by 0.5 mM KCl; for the K-deficient (DK) treatment, 9.4 mM KNO_3_ was replaced by 9.4 mM NaNO_3_ and 0.5 mM KH_2_PO_4_ was replaced by 0.5 mM NaH_2_PO_4_. At the same time of nutrient deficiency treatments, the wild-type *Arabidopsis* leaves were sprayed with 5 mmol/L vitamin E solution or 5 mmol/L vitamin C solution (every 3 days), representing the exogenous vitamin E treatment and the exogenous vitamin C treatment, respectively.

### 4.2. Determination of Chlorophyll Contents

On the 35th day and 45th day after germination (10 days and 20 days of nutrient deficiency treatments, respectively), the rosette leaves and seed pods were harvested for chlorophyll determination. For the leaves, 0.2 g materials were ground into 4 mL of acetone/ethanol (*v*/*v*, 1:1) mixture, and incubated for 24 h in the dark. For seed pods, 0.2 g materials were ground into powder by liquid nitrogen, 1 mL of acetone/ethanol (*v*/*v*, 1:1) mixture was added, vortexed and mixed for 30 s, then they were centrifuged at 4 °C, 8000 rpm for 10 min. The absorbance at wavelengths of 470, 646 and 663 nm of the supernatant was measured. The chlorophyll contents were calculated according to Porra et al. [56].

### 4.3. Determination of Ethylene Contents

On the 45th day after germination (20 days of nutrient deficiency treatments), the seedlings were harvested for ethylene determination. Ethylene estimation was performed according to Datta et al. [57]. Leaves were incubated in a 20-mL gas chromatographic box at 20 °C for 1 day. The box was flushed with ethylene-free air and sealed then. The ethylene level was detected with a gas chromatograph (GC-2010, Shimadzu Comp., Kyoto, Japan) equipped with a Pora-PLOT U column. The samples were injected into the column, which was pre-warmed at 30 °C. The ethylene content was quantified according to the standard sample.

### 4.4. Determination of Jasmonic Acid Contents

On the 45th day after germination (20 days of nutrient deficiency treatments), the seedlings were harvested for jasmonic acid determination. A total of 0.5 g leaves were ground into powder by liquid nitrogen and transferred into a 10 mL centrifuge tube, 2 mL of 80% methanol aqueous solution containing 0.5% formic acid was added, and the jasmonic acid was ultrasonically extracted at 4 °C for 30 min and then incubated overnight. After centrifuging at 12,000 rpm at 4 °C for 15 min, the supernatant was rotated and depressurized at 38 °C for evaporation and then frozen at −20 °C for 30 min. The thawing sample was centrifuged at 12,000 rpm at 4 °C for 10 min, the pigments and lipid insolubles were discarded, and then the supernatant was shaken vigorously for 30 s, and centrifuged at 4000 rpm for 5 min. The supernatant was concentrated and dried with a nitrogen blower, and reconstituted in 1.0 mL acetonitrile solution, ultrasonicated for 30 s, and filtered through a 0.22 μm microporous membrane. The liquid chromatography–TurboIon Spray tandem mass spectrometric (LC/MS/MS) analysis was performed on an Agilent 1100 LC equipped with an MDS/Sciex API 3000 triple-quadrupole mass spectrometer (MDS/Sciex, Thornhill, ON, Canada). The experimental parameters were set as Kristl et al. [58] described.

### 4.5. Quantitative Real-Time PCR

On the 45th day after germination (20 days of nutrient deficiency treatments), the seedlings were harvested for RNA extraction with the TRIzol™ Plus RNA Purification Kit (Invitrogen, Carlsbad, CA, USA). All RNA samples were treated with DNase I before RT-PCR. For each sample, 1 μg RNA was subjected to cDNA synthesis by using SYBR Premix Ex Taq (Takara Biotechnology Dalian Co., Ltd. Dalian, China). Then the quantitative PCR (qPCR) was performed with the EmeraldAmp MAX PCR Master Mix (Takara Biotechnology). The threshold cycle (*C*_t_), defined as the PCR cycles when the product could be first detected, was measured to the determine relative expression levels of target genes [59]. Three biological replicates with three technical repetitions were performed for each sample. *ACTIN7* gene was used as an internal control. Normalization of qPCR data was achieved by subtracting the *C*_t_ value of the internal reference gene from the *C*_t_ values of the target genes to get Δ*C*_t_ [60]. The individual gene expression levels were presented as the fold-changes relatively to *ACTIN7* expression levels (2^−^^Δ*C*t^). The expression level of the WT in the control sample (CK) was normalized into “1.0”. All primers are listed in Table 1.

### 4.6. Oxidative Damage Measurements

Histochemical staining for hydrogen peroxide or superoxide in 45-day-old seedlings (20 days of nutrient deficiency treatments) was performed by incubation with 3,3’-diamino-benzidine (DAB) or nitro-blue tetrazolium (NBT), respectively, for 2–4 h according to the procedure as previously described [61,62]. Stained leaves were boiled in 80% ethanol for decoloration of chlorophylls and scanned.

The quantitative level of O_2_^−^ was detected with the method as described previously [63] via monitoring the NH_2_OH to NO_3_^−^ conversion. The quantitative level of H_2_O_2_ was detected with the method as described previously [64] through a chromogenic reaction with trichloroacetic acid (TCA). The level of oxidative damage to the cytomembrane (lipid peroxidation) was assessed by detecting the malondialdehyde (MDA) concentrations with the method as described previously [65]. Electrolyte leakage was measured by a conductivity meter (DDSJ-319L, INESA Scientific Instrument Co., Ltd., Shanghai, China).

### 4.7. Germination Rate Determination

After harvesting the *Arabidopsis thaliana* seeds under each treatment condition, the seeds were vernalized for 2 days under dark conditions at 4 °C. Then the seeds were disinfected with 75% ethanol and 0.1% mercury (HgCl_2_ solution) on an ultraclean workbench. The seeds were sowed evenly (1 cm apart) on 1/2 MS medium, and sealed with parafilm to ensure that the seeds grew under aseptic conditions. The sealed medium was transferred to a light culture room for cultivation (temperature 23 °C; humidity 70%; light intensity 100 μmol·m^−2^·s^−1^; photoperiod 16 h light/8 h dark), and the number of seeds germinated after 5 days was counted.

### 4.8. Statistical Analysis

A total of 3-5 independent replicates were conducted for each measurement. The statistical analysis was carried out with the software SPSS 19.0 (IBM Comp., Chicago, IL, USA). The Duncan’s multiple range test was performed to compare the means. *p* < 0.05 was used to estimate a statistically significant difference. Error bars in the figures represent the standard error of the mean (SEM).

## Figures and Tables

**Table 1:** The PCR primers sequences.

Gene	Locus	The Forward Primer Sequences	The Reverse Primer Sequences
*VTC1*	At2g39770	GGCAACCCCGTGACTACATAAC	CCAATCAAACATCCTTCCCCAA
*VTC2*	At4g26850	GGTCGTCACTTGAAGAAGAGGC	GGGAAGAACTGAACTTGGGCAT
*VTE4*	At1g64970	AGCAGCACCCTCTTCTCTCACA	CCCAAATCTCTTCCCACAAACC
*GGR*	At4g38460	ATGGTGGAGCAGAGAAGGGAAT	AGGTGGTAGCGAAGATGAATGG
*ACS2*	At1g01480	GTGTCTCCTGGCTCTTCCTTCC	GCCGTCAAAAACAACCCTAATG
*ACO1*	At2g19590	TCCTGAGCTTATGAGAGGGCTG	AATGGTATTGTTCTTGGATGGC
*EIN3*	At3g20770	ACAACAATAACAGTAGCGGCAACA	AGCGATAGAGACAGAGAGACCCAG
*ERF1*	At3g23240	GCAGTCCACGCAACAAACCTA	CTTGAACTCTCTCCGCCGAAA
*PDF1.2*	At5g44420	CTTGTTCTCTTTGCTGCTTTCG	CATGATCCATGTTTGGCTCCTT
*COL1*	At5g15850	AATGGCTTCTCGATTGGGGAT	TGGAGGGTAAGGTGGTTGGTC
*ACT7*	At5g09810	ATCCCTCAGCACCTTCCAAC	ACCCGATACTTAAATAATTGTCTCAT

VTC1, Vitamin C defective 1; VTC2, Vitamin C defective 2; VTE4, Vitamin E defective 4; GGR, Geranylgeranyl Reductase; ACS2, 1-Aminocyclopropane-1-Carboxylic acid Synthase 2; ACO1, 1-Aminocyclopropane-1-Carboxylate Oxidase 1; EIN3, Ethylene Insensitive 3; ERF1, Ethylene Response Factor 1; PDF1.2, Plant Defensin 1.2; COL1, CONSTANS-Like 1; ACT7, ACTIN 7.
